# User-Centered Development of a Mobile App for Biopsychosocial Pain Assessment in Adults: Usability, Reliability, and Validity Study

**DOI:** 10.2196/25316

**Published:** 2021-05-14

**Authors:** Filipa Lopes, Mário Rodrigues, Anabela G Silva

**Affiliations:** 1 School of Health Sciences, University of Aveiro Aveiro Portugal; 2 Higher School of Technology and Management of Águeda, Institute of Electronics and Telematics Engineering of Aveiro (IEETA), University of Aveiro Aveiro Portugal; 3 Center for Health Technology and Services Research (CINTESIS.UA), School of Health Sciences, University of Aveiro Aveiro Portugal

**Keywords:** pain assessment, mobile app, validity, reliability, usability, mHealth, pain, user-centered design

## Abstract

**Background:**

Pain-related mobile apps targeting pain assessment commonly limit pain assessment to pain behaviors and physiological aspects. However, current guidelines state that pain assessment should follow the biopsychosocial model, clearly addressing biological, psychological, and social aspects of the pain experience. Existing reviews also highlight that pain specialists and end users are not commonly involved in the development process of mobile apps for pain assessment, negatively affecting the quality of the available apps.

**Objective:**

This study aimed to develop a mobile app for pain assessment (AvaliaDor) and assess its usability, validity, reliability, and measurement error in a sample of real patients with chronic pain recruited from a physiotherapy clinic.

**Methods:**

This study was divided into 2 phases: phase 1—development of the AvaliaDor app; and phase 2—assessment of the apps’ usability, reliability, measurement error, and validity. AvaliaDor was developed (phase 1) based on the literature and the recommendations of physiotherapists and patients with pain in cycles of evaluation, inclusion of recommendations, and reevaluation until no further changes were required. The final version of the app was then tested in patients with musculoskeletal pain attending a private physiotherapy practice (phase 2) who were asked to use the app twice on 2 consecutive days for reliability purposes. In addition, participants had to complete a set of paper-based scales (Brief Pain Inventory, painDETECT, Pain Catastrophizing Scale, and Tampa Scale for Kinesiophobia), which were used to assess the validity (criterion validity and hypothesis testing) of the app, and the Post-Study System Usability Questionnaire was used to assess its usability.

**Results:**

The development process (phase 1) included 5 physiotherapists external to the research team and 5 patients with musculoskeletal pain, and it resulted in the creation of an app named AvaliaDor, which includes an assessment of pain intensity, location, and phenotype; associated disability; and the issues of pain catastrophizing and fear of movement. A total of 52 patients with pain (mean age 50.12 years, SD 11.71 years; 39 females) participated in phase 2 and used the app twice. The Pearson correlation coefficient between the scores on the paper-based scales and the app ranged between 0.81 and 0.93 for criterion validity and between 0.41 and 0.59 for hypothesis testing. Test-retest reliability was moderate to good (intraclass correlation coefficient between 0.67 and 0.90) and the score for usability was 1.16 (SD 0.27), indicating good usability.

**Conclusions:**

A mobile app named AvaliaDor was developed to assess the intensity, location, and phenotype of pain; associated disability; and the issues of pain catastrophizing and fear of movement in a user-centered design process. The app was shown to be usable, valid, and reliable for assessing pain from a biopsychosocial perspective in a heterogeneous group of patients with pain. Future work can explore the long-term use of AvaliaDor in clinical contexts and its advantages for the assessment and management of patients with pain.

## Introduction

Currently, there is a rocketing expansion in the development of mobile apps all over the world, largely due to the increased accessibility and global availability of smartphones [1]. The technology of mobile devices has greatly improved in recent years, with higher screen resolution and better processor performance, among other improvements in hardware. Furthermore, using smartphones to access the internet is part of everyday life [2], establishing a technological revolution. This advancement in digital technology is also changing health care [3] and making it more accessible [1].

The use of information and communication technologies in health is called eHealth [4]. According to the World Health Organization, mobile health (mHealth)—which covers medical and public health practices supported by mobile devices, such as mobile phones, user monitoring devices, personal digital assistants, and other wireless devices [5]—is a component of eHealth. Among the advantages of mHealth is its use of smartphones instead of traditional personal computers, which provides self-monitoring support to the user in most everyday situations [6,7]. mHealth facilitates access to information related to the user’s health conditions or treatments, allows the organization and recording of health information, allows for self-monitoring and self-management, and facilitates communication between patients and health care providers and interaction between health care users or providers and health services registration systems [8]. Also, eHealth has been shown to overcome current health care problems and limitations, such as difficulties accessing timely and continuous health care, the associated costs, mobility limitations, or long wait times [9,10]. This is particularly relevant for chronic conditions, such as chronic pain, in which self-management is a key component of the intervention.

Pain-related mobile apps targeting pain assessment commonly include electronic diaries to monitor pain characteristics such as intensity, location, factors that aggravate or alleviate pain, and/or intake of medication [8,11,12], largely limiting pain assessment to pain behaviors and physiological aspects and not including a biopsychosocial assessment of pain [8]. A systematic search found 142 pain-related apps, of which 28 were primarily intended for pain assessment [8]. However, when comparing the existing mobile apps against the existing data on their development, it was also found that data on reliability, validity, and usability were scarce for pain assessment apps [8]. Another study corroborated these findings by concluding that despite the large and growing number of existing mobile apps related to pain, their quality is seldom guaranteed [13]. This may be due, in part, to the lack of involvement of health professionals and users in the process of developing the app [14]. This involvement is a way of ensuring the validity of the content of the mobile app [15] and that it meets the users’ needs and requirements [16], increasing its likelihood of being usable. Usability is an essential criterion in the evaluation of mHealth apps [17,18] and has been defined as the extent to which a product can be used by users to achieve specific goals with effectiveness, efficiency, and satisfaction and in a specific context of use [19]. Nevertheless, a systematic review that synthesized and evaluated existing studies on the assessment of the usability of pain-related apps found 31 manuscripts on the usability of 32 pain apps [20]. The results of this systematic review suggested that several important methodological aspects regarding the assessment of usability are not being considered when developing pain-related apps, such as not using reliable and valid instruments to assess usability [20].

As measurement instruments, mobile apps that intend to be used for pain assessment need to provide evidence of their reliability, measurement error, and validity. Reliability refers to the consistency of results in repeated measurements performed in similar circumstances [21]. Measurement error informs on the amount of change needed between successive measurements that could be considered a real change in the person’s condition [16]. Validity refers to whether an instrument measures what it intends to measure [21]. The main aim of this study was to develop a mobile app for the biopsychosocial assessment of pain and to assess it for usability, validity, reliability, and measurement error in a sample of real patients with chronic pain recruited from a physiotherapy clinic. The app was developed in a user-centered design paradigm, involving the users from the very beginning.

## Methods

This study describes the development of a mobile app for pain assessment and its usability, validity, reliability, and measurement error assessment. It received ethical approval from the Council of Ethics and Deontology of the University of Aveiro. All participants provided written informed consent before entering the study.

### Procedures

This study was divided into 2 phases: phase 1—development of the app; and phase 2—assessment of the app in terms of its usability, reliability, measurement error, and validity. The development of the app (phase 1) included the (1) validity of the app’s content, (2) content analysis and user interface design, (3) prototype development, (4) prototype testing, and (5) prototype modification and construction of the final app. These phases and subphases are described below.

### Phase 1: Development of the App

#### Validity of the App’s Content

The first step in the development of the app was to define the fundamental aspects that should be considered for a biopsychosocial pain assessment. This was based on the literature on pain assessment, existing mobile apps for pain assessment, and input from physiotherapists and patients with chronic pain. First, we considered the recommendations from the Initiative on Methods, Measurement, and Pain Assessment in Clinical Trials (IMMPACT) on the pain-related domains that should be assessed for patients with chronic pain [22] and analyzed existing apps, such as Keele Pain Recorder [23] and Pain Monitor [24]. Based on this analysis, and previous experience and knowledge, the 2 physiotherapists on the research team (FL and AGS) defined the preliminary contents that the app should cover: pain intensity, pain location, impact of pain, influence of pain on sleep, pain catastrophizing, fear of movement, and main mechanism of pain (eg, nociceptive or neuropathic). Then, validated instruments that covered most of the defined domains were identified and adapted to be included in the app: the Brief Pain Inventory-Short Form [25,26], covering pain intensity, disability, and sleep; and the painDETECT questionnaire [27], covering the phenotype of pain. Both scales have a body map for pain location. Also, catastrophizing and fear of movement were assessed in the mobile app with a single question on each of the constructs, retrieved from the Pain Catastrophizing Scale and the Tampa Scale for Kinesiophobia, respectively. The questions were identified based on their predictive ability [28,29].

#### Content Analysis and User Interface Design

Once the information to be included in the app was determined, paper-based mock-ups were produced using Adobe XD (Adobe Inc) to visualize the information displayed in a format similar to that of the proposed mobile app. These mock-ups were shown to 5 physiotherapists—experts in musculoskeletal physiotherapy—and 5 patients with musculoskeletal pain. Physiotherapists had to have a master’s degree or be enrolled in their second year of a master’s degree program in the area of musculoskeletal physiotherapy and have a minimum of 2 years of professional experience in musculoskeletal physiotherapy. Patients were included if they were aged ≥18 years, reported musculoskeletal pain, were able to speak and read Portuguese, and understood the main objectives of the study. Both physiotherapists and patients were asked the following questions: (1) Do you think the app covers all relevant content for pain assessment?; (2) Are the questions clear and concise?; (3) Are the response options adequate?; (4) Is the order of the questions the most appropriate?; and (5) Do you have any suggestions for improvement? All interviews were recorded, and notes were taken regarding suggested changes. We found that both patients and physiotherapists provided similar recommendations, and no new information was added after the fifth participant of each group (we reached theoretical saturation); thus, no further participants were recruited at this stage. The sample size for the development phase of the app was informed by data saturation. This is a principle widely accepted in qualitative research and indicates that, based on the data that have been collected, further data collection is unnecessary [30].

#### Prototype Development

The prototype was developed for the Android operating system using Android Developer Studio (Google, Inc). First, we implemented the screen layouts according to the mock-ups and tested them with distinct screen sizes, ratios, and pixel densities for mobile phones. Adjustments were made for better fits, and all adjustments were validated by the mock-up’s designer. After layout validation, the elements were made functional and the dynamic look and feel were also validated by the mock-up’s designer.

At the end of each registration, the data were sent via the internet to a server featuring a back office for data visualization and download. Each record was a tuple containing all collected answers plus images of the marked body maps in PNG format. If a network connection was not available, the data were stored locally—the tuple in an SQLite database and the images in the app-specific folder. Whenever locally stored records existed, the user was informed on the first screen of the app and he/she could choose to send them. The records sent were then erased from the local database and file system.

#### Prototype Testing: Preliminary Usability Evaluation

The information gathered in previous steps was used to inform the first version of the app—AvaliaDor, version 1. This version of the app was tested by the same 5 physiotherapists and 5 patients that had analyzed the mock-ups. They were interviewed once more after having used the app in a preliminary usability evaluation. The physiotherapists were given the app 1 week before the interview. Both physiotherapists and patients were asked the following questions: (1) Do you think the app is functional?; (2) Do you think it is simple to use?; (3) Do you notice any aspect that should be improved ?; (4) On what model of mobile phone did you use the app?; and (5) Was the app well-adjusted to the screen? In addition, the time taken to complete a full pain assessment using the app and any errors were registered (performance indicators for usability evaluation).

#### Prototype Modification and Construction of the Final App

The physiotherapists in the team and the programmer discussed the suggestions made in the previous step of app development, and another version of the app—AvaliaDor, version 2—was developed and tested again by the same 5 patients. Physiotherapists were not included at this stage because they made only minor recommendations for improvement in the prototype testing subphase. Once no further changes were recommended by patients, the AvaliaDor, version 2, became the final version of the app.

### Phase 2: Assessment of the App in Terms of its Usability, Reliability, Measurement Error, and Validity

#### Participants and Instruments

Participants were patients with musculoskeletal pain attending a private physiotherapy practice. To enter the study, they were required to report pain in the limbs or back, be aged ≥18 years, be able to read and speak Portuguese, and understand the aims of the study (assessed by asking participants to explain in their own words what the study was about). The sample size was based on indicators for reliability studies. To attain a maximum intraclass correlation coefficient (ICC) of 0.8—with 2 measurements, an alpha of 5%, and a power at 80%—at least 46 participants were needed [31].

Participants were asked to use the AvaliaDor app to assess their pain on 2 consecutive days for reliability assessment purposes. On the second day, before using the app, patients were asked if there were changes in their pain since the previous day. Those answering yes to this question were excluded from the reliability analysis. Also, in the first session, patients were asked to complete the following questionnaires, which were used to assess hypothesis testing and criterion validity:

- User characterization questionnaire—this questionnaire collected data on gender, age, educational qualifications, marital status, current clinical diagnoses, and duration of pain.
- Pain Catastrophizing Scale—consists of 13 items that depict thoughts, perceptions, or feelings associated with pain [32,33]. Participants are asked to indicate to what extent they present with the thoughts and feelings described using a 4-point Likert scale (0=never to 4=always). The final score represents the sum of all items and varies between 0 and 52, with higher scores reflecting higher levels of catastrophic thoughts [34,35]. The ICC for the reliability of the Portuguese version of this scale varies between 0.78 and 0.82 [32].
- Tampa Scale for Kinesiophobia—consists of 13 items that measure kinesiophobia under generic conditions. Each item is scored on a 4-point Likert scale, from 1 (strongly disagree) to 4 (strongly agree). The total score corresponds to the sum of the scores obtained for each of the items and varies from 13 to 52 points, where 13 represents the lowest and 52 the highest degree of kinesiophobia [36]. It showed good levels of test-retest reliability (ICCs between 0.94 and 0.98) and internal consistency with a Cronbach α of 0.82 [36].
- Brief Pain Inventory—consists of 2 subscales: the pain severity and pain interference subscales [25,32]. The score for pain severity ranges from 0 to 40 and the score for pain interference ranges from 0 to 70 [37]. This instrument has excellent internal consistency levels with Cronbach α values for the pain severity and interference subscales of 0.99 and 0.84, respectively. Its test-retest reliability also has very satisfactory values: ICCs of 0.88 for pain severity and 0.84 for pain interference [32].
- painDETECT questionnaire—aims to identify the nociceptive, neuropathic, or mixed component of the pain phenotype [27]. Its score ranges from 0 to 38, where 0 to 12 corresponds to nociceptive pain, 13 to 18 indicates a mixed pain, and 19 to 38 corresponds to neuropathic pain. This questionnaire has been shown to be reliable [38].

Also, further assessment of the app’s usability was conducted through the Post-Study System Usability Questionnaire. This questionnaire was developed to assess users’ satisfaction with the usability of a digital solution. It consists of 19 items that can either be scored using an 8-point Likert scale, anchored at the lower end with “totally agree” and at the higher end with “totally disagree,” or be scored as not applicable. The total score is the mean of all items and lower scores are indicative of better usability. The Portuguese version has good internal consistency with a Cronbach α of 0.80 and acceptable interrater reliability (ICC 0.67) [39].

#### Statistical Analysis

SPSS software (version 26; IBM Corp) was used to perform data analysis. For sample characterization, the mean and standard deviation were used for continuous variables, while frequency distribution and percentage were used for ordinal and nominal variables. The criterion validity of the app questions on pain location, pain intensity, pain interference, and pain phenotype was assessed by correlating the app scores with the paper-based scores of the respective paper-based questionnaire using the Pearson correlation coefficient. Criterion validity was considered to exist when the correlation between the results of the app and the respective gold standard (paper-based questionnaire) was ≥0.7 [40]. For the questions on fear of movement and catastrophizing, construct validity was assessed by correlating the scores obtained using the app with the scores obtained on the paper-based Tampa Scale for Kinesiophobia and Pain Catastrophizing Scale, respectively, also using the Pearson correlation coefficient. For construct validity, the correlation coefficient was interpreted as indicating correlation—low (<0.3), moderate (0.3-0.5), and strong (>0.5) [41]—and we hypothesized that there would be a moderate correlation between the app scores and the paper-based scores.

Test-retest reliability was assessed using the ICC (bidirectional randomness, absolute agreement) and the respective 95% confidence interval. The results were interpreted as weak (ICC <0.50), moderate (ICC 0.50-0.75), good (ICC 0.75-0.90), and excellent (ICC ≥0.90) [42]. In addition, measurement error was assessed using the standard error of measurement (SEM) and the minimal detectable difference (MDD) with a confidence level of 95% (MDD95), calculated as SEM = SD√ (1-ICC) and MDD95 = SEM × 1.96 × √2 [43]. We also calculated the limits of agreement and constructed Bland-Altman plots [44] both for the criterion validity and for the test-retest reliability.

## Results

### Phase 1: Development of the AvaliaDor App

The most relevant characteristics of the patients with pain and the physiotherapists who contributed to the process of development and analysis of the app are presented in Tables 1 and 2, respectively.

**Table 1:** Characteristics of patients involved in the development phase (phase 1) of the mobile app (n=5).

Variable		Number of patients
**Gender**		
	Female	4
	Male	1
**Age (years)**		
	18-30	1
	31-50	2
	51-60	1
	61-70	1
**Education level (school years)**		
	6	1
	9	1
	12	1
	University	2
**Location of main pain complaint**		
	Neck	1
	Knee	1
	Hip	1
	Face	1
	Low back	1

**Table 2:** Characteristics of the physiotherapists involved in the development phase (phase 1) of the mobile app (n=5).

Variable			Number of physiotherapists
**Gender**			
	Female	3	
	Male	2	
**Age (years)**			
	25-30	2	
	31-40	3	
**Experience as a physiotherapist (years)**			
	5-9	3	
	10-12	2	

The main suggestions of the physiotherapists regarding the mock-ups included creating a final summary report of the assessment, changing the caption of the body map, and adding an option regarding the location of the pain instead of featuring a body map. Only 1 patient suggested changes to the app, namely to change the order of the questions. Table 3 lists the patients’ and physiotherapists’ suggestions and comments, as well as the resulting decisions made by the research team.

**Table 3:** Summary of physiotherapists’ and patients’ comments on the mock-ups of the AvaliaDor app.

Question	Physiotherapists’ comments	Patients’ comments	Decisions made by the research team
Do you think the app covers all the relevant content for pain assessment?	All physiotherapists found that the app covered all relevant content, apart from 1 physiotherapist who suggested including injury mechanism, pain duration, relief, and/or worsening factors.	All patients stated that everything relevant was included.	As most physiotherapists and all users reported that the app covered the most relevant content, no further contents were included.
Are the questions clear and concise?	All physiotherapists stated that the questions were clear and concise. One physiotherapist noted that in the question, “Does your pain spread to other regions of the body?”, an affirmative answer required the user to go back, and this should be amended.	All patients reported that the questions were clear and concise.	A response option was added following the question, “Does your pain spread to other regions of the body?”
Are the response options adequate?	Two physiotherapists, despite claiming that the response options were adequate, suggested that it should be possible for users to mark the exact area of pain on the body map and that response options should be wider apart so that the different options are clearer.	All users reported that the answers were adequate. One user even mentioned that the examples were very enlightening and illuminating.	Both of the physiotherapists’ suggestions were included in the app.
Is the order of questions the most appropriate?	All physiotherapists agreed that the order was the most appropriate.	All but 1 patient agreed on the order of the questions. One patient suggested that the questions on pain interference should be listed before the questions on pain intensity and pain location.	As all physiotherapists and most patients agreed with the order of the questions, which is also aligned with what is more common in existing apps and scales, no change was made.
Do you have any suggestions for improvement?	A number of suggestions were made: include a final report with a summary score; calculate the average pain from the worst and least pain intensities; precede the question on the effect of the treatment with a question on whether patients were receiving treatment; include “injury mechanisms,” pain duration, and “relieving/aggravating factors”; change the body map caption to include both chief pain complaint and other pain complaints; and check the verb tenses so that all questions use the same tense.	No suggestions for improvement were made.	A summary report was included; the question on the average pain intensity was maintained in line with the original version of the questionnaire; and, for a similar reason, a question on whether patients were receiving treatment was not included. The team decided not to include questions on the mechanism of injury, duration of pain, and relieving/aggravating factors because it would make the app questionnaire too extensive. The legend of the body map was modified in line with physiotherapists’ comments, and the verb tenses were corrected.

The first version of the app was then developed and again shown to users for assessment. Physiotherapists’ and patients’ general opinion of the app was quite favorable, reporting that it was functional and easy to use. Criticisms and suggestions from physiotherapists and users are presented in Table 4. Table 4 also shows the models of the mobile phones used to run the app because the characteristics of the screen have an impact on the readability and activation of the response options, and the type of processor influences the fluidity of the rendering of the body map. Participants took between 5 and 8 minutes to complete all of the steps for pain assessment in the app, and no app-related errors were identified.

Next, a second version of the app was developed, and patients reported that no further changes were required.

The final version of the AvaliaDor app includes the following:

- a login page;
- a body map (front, back, right side, and left side) for the patient to draw pain, pins and needles, numbness, or another symptoms;
- questions on pain phenotype based on the painDETECT questionnaire;
- questions on pain intensity (at present, at its worst, and over the last week);
- questions on pain interference based on the Brief Pain Inventory;
- 1 question on fear of movement;
- 1 question on pain catastrophizing; and
- a report generated with the patient’s data in the form of a Microsoft Excel file.

**Table 4:** Summary of physiotherapists’ and patients’ comments on the first version of the AvaliaDor app.

Commenter (models of mobile phone) and suggestions		Decisions made by the research team
**Physiotherapists (Samsung Galaxy S7; Samsung Galaxy S6; Huawei P10 Lite; Huawei P20)**		
	The app was considered easy to use except for the body map, which might be too small for patients to mark different symptoms on (n=2).	The size of the body map was increased.
	It was suggested that a question asking whether the patient felt some relief from their symptoms should be included before asking the patient to indicate the extent of that relief (n=2).	No question was added, as it was believed that the patient could indicate 0% improvement if no relief from symptoms was experienced.
	The body map’s legend should state “main pain” instead of “pain” to minimize confusion if the patient had more than one painful body site (n=2).	The body map’s legend was changed accordingly.
	There should be a “go back” sign (n=1).	An arrow was added to make it clearer how to go back.
	The font size might be small for older adults (n=1).	No change to the font size was made, as it was not identified by the older patients in the sample as being a limitation.
**Patients (Samsung Galaxy S7)**		
	It was difficult to draw the pain area (n=2).	The size of the body map was increased.
	The researcher was asked how someone should answer the question about pain relief in the case that no treatment was being received (n=2).	No question was added, as it was considered that the patient could indicate 0% improvement if no pain relief was experienced.

**Figure 1:**
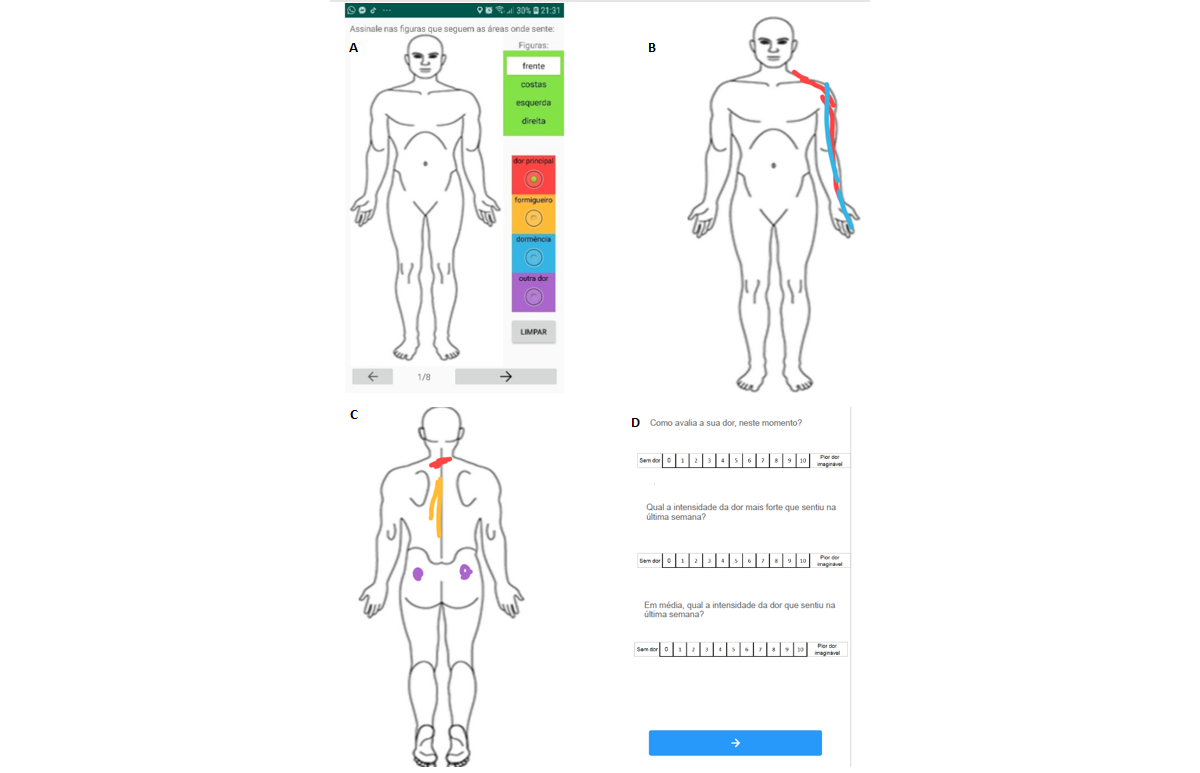
Print screens from the app. (A) Front body map. Front (B) and back (C) body maps after being filled in by the patient. (D) List of questions.

Figure 1 shows the following screen excerpts from the app: the body map (Figure 1A); filled body maps (Figure 1B and 1C); and a page of questions (Figure 1D).

### Phase 2: Usability, Reliability, Measurement Error, and Validity

The study sample consisted of 52 participants aged between 22 and 72 years (mean 50.12 years, SD 11.71 years). Table 5 presents a more detailed description of the sample.

**Table 5:** Characteristics of patients who participated in the assessment of the final version of the app (n=52).

Characteristics		Values, n (%)
**Gender**		
	Female	39 (75)
	Male	13 (25)
**Age (years)**		
	<50	23 (44)
	≥50	29 (56)
**Education level (school years)**		
	4	5 (10)
	6	19 (37)
	9	12 (23)
	12	4 (8)
	University	12 (23)
**Location of main pain complaint**		
	Neck	6 (12)
	Low back	8 (15)
	Shoulders	17 (33)
	Elbows	4 (8)
	Hands	3 (6)
	Hips	1 (2)
	Knees	4 (8)
	Legs	1 (2)
	Feet	8 (15)
**Pain duration**		
	<3 months	7 (13)
	≥3 to <6 months	8 (15)
	≥6 months to <1 year	2 (4)
	≥1 to <2 years	12 (23)
	≥2 to <5 years	8 (15)
	≥5 years	15 (29)

#### Usability Evaluation

Usability was tested using the Post-Study System Usability Questionnaire, and the average score obtained was 1.16 (SD 0.27), indicating that the app was considered usable.

#### Reliability and Measurement Error

**Figure 2:**
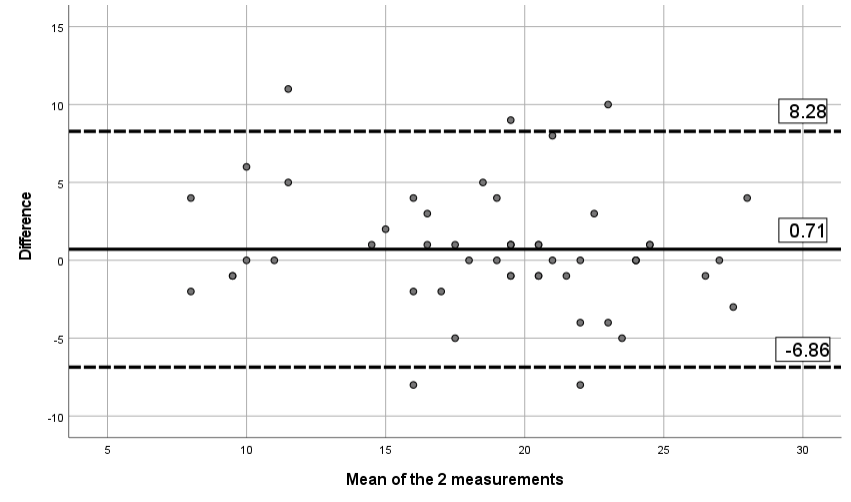
Bland-Altman limits for the Brief Pain Inventory severity subscale between the 2 assessments using the app.

**Figure 3:**
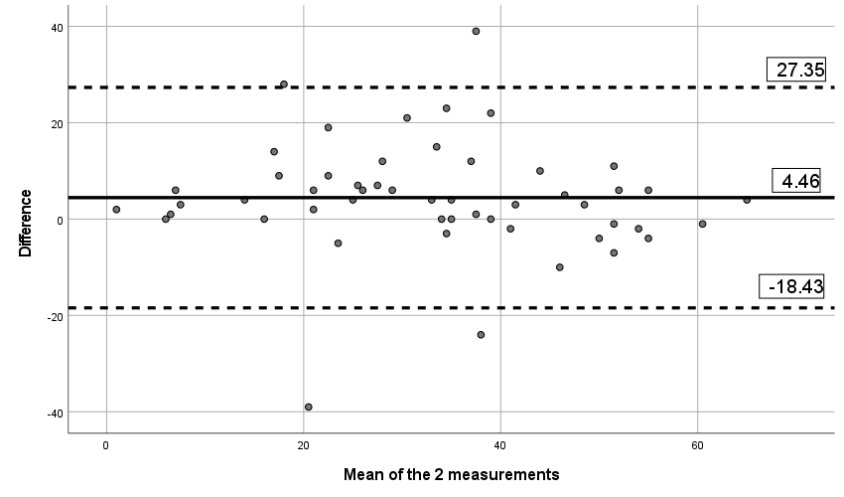
Bland-Altman limits for the Brief Pain Inventory pain interference subscale between the 2 assessments using the app.

**Figure 4:**
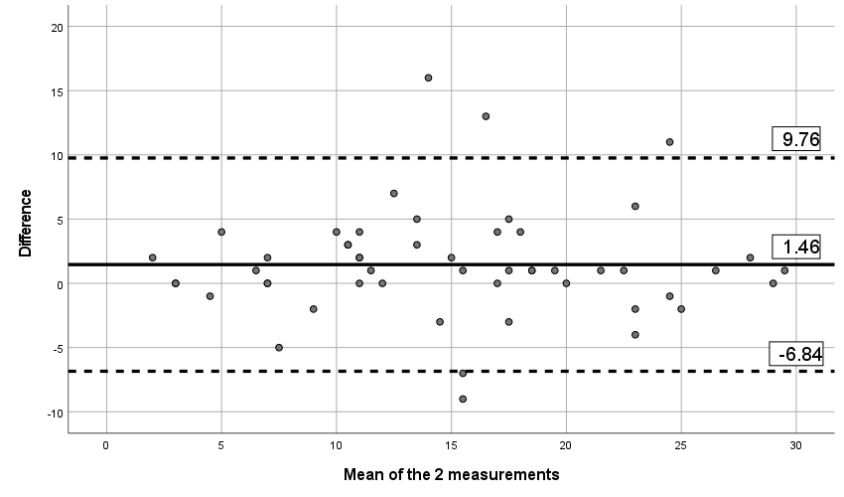
Bland-Altman limits for the painDETECT questionnaire between the 2 assessments using the app.

**Figure 5:**
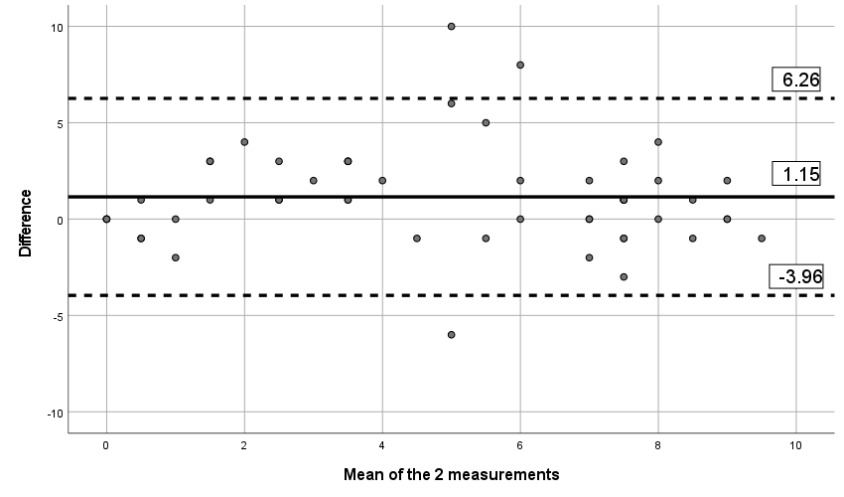
Bland-Altman limits for the issue of pain catastrophizing between the 2 assessments using the app.

**Figure 6:**
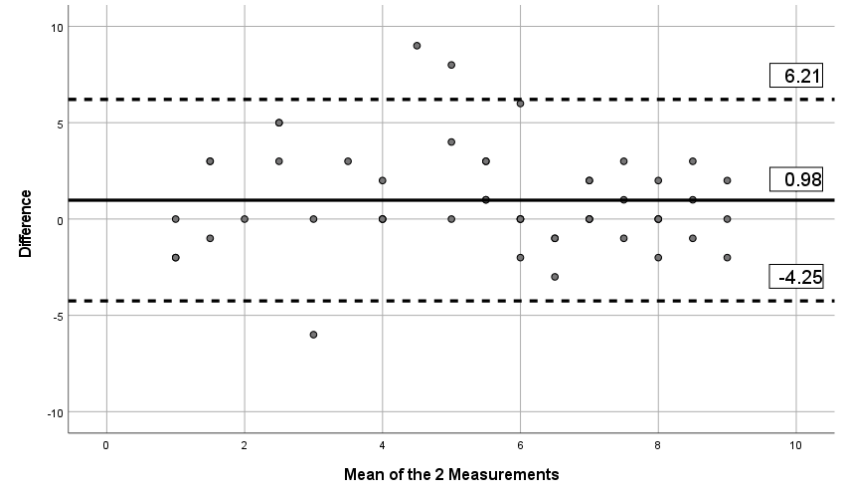
Bland-Altman limits for the issue of fear of movement between the 2 assessments using the app.

**Figure 7:**
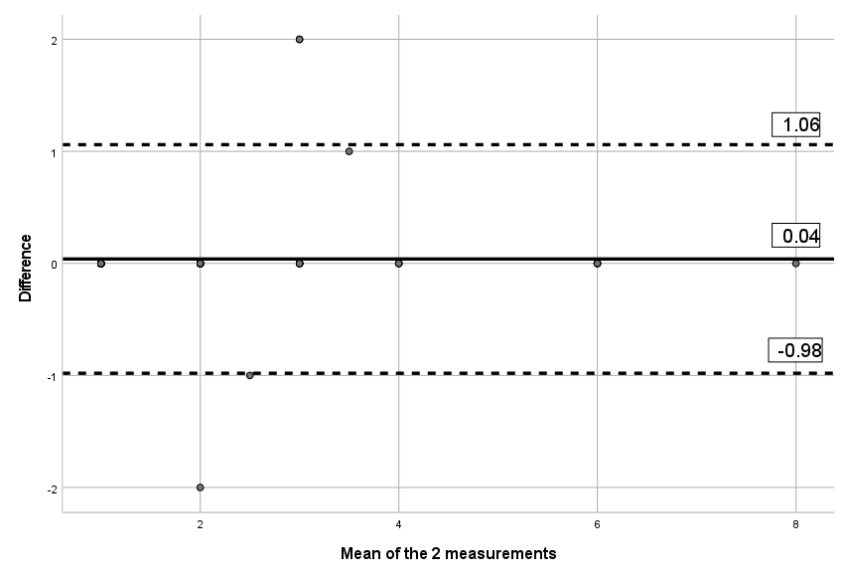
Bland-Altman limits for the number of painful body sites between the 2 assessments using the app.

The ICCs obtained between the 2 assessments and the mobile app indicates moderate to excellent reliability, with ICCs between 0.67 and 0.90, as presented in Table 6. Table 6 also presents the SEM and MDD. Visual inspection of the limits of Bland-Altman plots (Figures 2-7) shows a symmetrical distribution around the mean, close to 0, with no systematic or proportional bias.

**Table 6:** Test-retest reliability and standard error of measurement of the AvaliaDor app.

Variable	Intraclass correlation coefficient (95% CI)	Standard error of measurement	Minimal detectable difference
Pain severity	0.86 (0.76-0.92)	1.96	5.43
Pain interference	0.84 (0.71-0.91)	6.40	17.73
Pain phenotype	0.90 (0.82-0.95)	2.35	6.52
Pain catastrophizing	0.76 (0.57-0.88)	1.30	3.60
Fear of movement	0.67 (0.42-0.81)	1.79	4.97
Number of painful body sites	0.98 (0.97-0.99)	0.24	0.66

#### Criterion Validity Assessment

**Figure 8:**
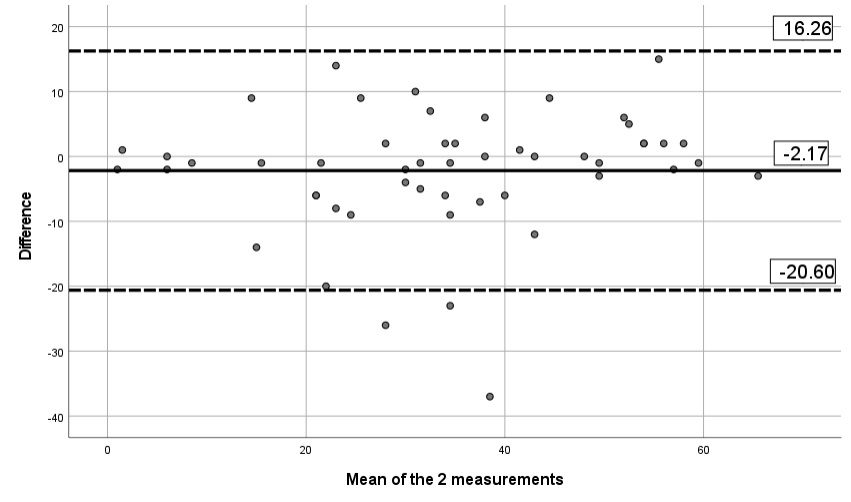
Bland-Altman limits for the Brief Pain Inventory pain interference subscale using the paper questionnaire and the mobile app.

**Figure 9:**
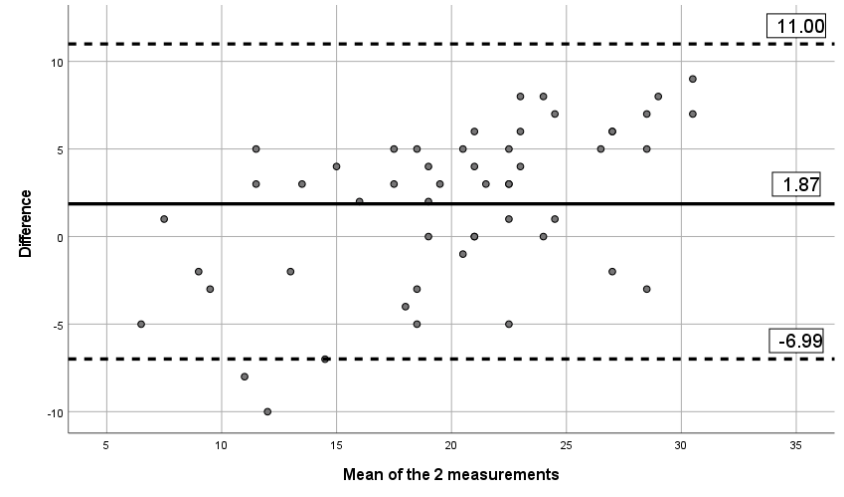
Bland-Altman limits for the Brief Pain Inventory pain severity subscale using the paper questionnaire and the mobile app.

**Figure 10:**
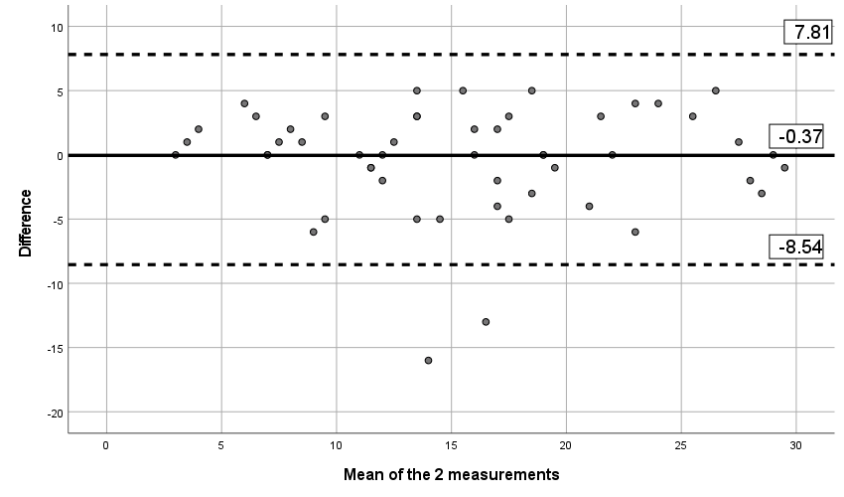
Bland-Altman limits for painDETECT using the paper questionnaire and the mobile app.

**Figure 11:**
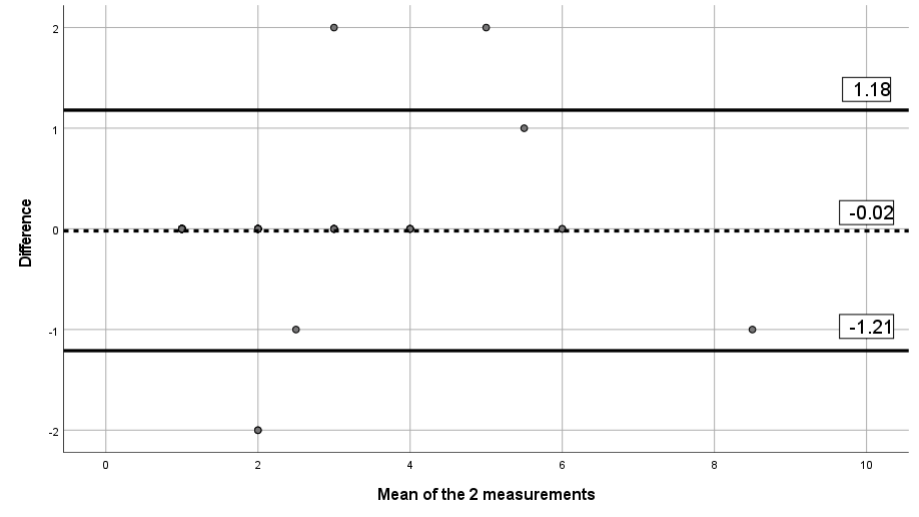
Bland-Altman limits for the number of painful body sites using the paper questionnaire and the mobile app.

The correlation between the paper version of the questionnaire and the app version was 0.84 for pain severity, 0.80 for pain interference, 0.84 for pain phenotype, and 0.93 for the number of painful body sites. Besides, visual inspection of the Bland-Altman limits (Figures 8-11) shows a symmetrical distribution around the mean, close to 0, with no systematic or proportional bias.

#### Hypothesis Testing Assessment

The correlation between the Pain Catastrophizing Scale administered on paper and the single question on catastrophizing included in the app was *r*=0.59 (*P*<.001), indicating a strong correlation. The correlation between the Tampa Scale for Kinesiophobia and the single question on fear of movement included in the app was *r*=0.41 (*P*=.003), indicating a moderate correlation.

## Discussion

### Principal Findings

This paper presents the process of development of an app that aims to assess pain from a biopsychosocial perspective. The app assesses the intensity, location, and phenotype of pain; the associated disability; and the issues of pain catastrophizing and fear of movement. It was developed in close collaboration with patients and physiotherapists from a user-centered perspective. The results of 2 assessments in 2 different sessions were promising and suggest that the developed app (AvaliaDor) is valid, reliable, and usable.

A previous review reported that of 283 pain-related apps that were available in the main shops (eg, App Store and Google Play), none has undergone a scientific process of validation [13]. This finding undermines the trust that both patients and health professionals have in these solutions, compromising the potential and added value that these solutions can bring to the care of patients with pain. For example, mobile apps can capture real-time data (reducing the impact of memory bias on the outcomes), help clinicians reach a larger number of patients [13], and help detect a deterioration in the pain condition that may alert the clinician to schedule an appointment. However, the development of mobile apps through a user-centered and scientifically sound process is of utmost importance for the clinician to trust its results.

The development process of our app was based on guidelines of which aspects of pain should be assessed [22] but also on previous apps that were considered good examples and on the feedback of physiotherapists who were experts on pain assessment and treatment. This guaranteed that the app covered the relevant aspects of pain that need to be assessed to inform the treatment of patients with pain and to assess the evolution of these patients with treatment (ie, guarantees content validity). Also, the high correlation values between the paper versions of the questions for pain severity, pain impact, pain location, and pain phenotype and the same measurements using the AvaliaDor app shows that the app has criterion validity (ie, that the measures taken with the app are an adequate reflection of the “gold standard” paper-based questionnaires). The Bland-Altman plots supported these findings with small mean differences between the 2 methods of measurement (app versus paper-based questionnaires). The correlations between 1 question on pain catastrophizing and 1 question on fear of movement with the full paper-based Pain Catastrophizing Scale and the Tampa Scale for Kinesiophobia, respectively, were in line with the predefined hypothesis and suggest that the questions used in the app were measuring the respective constructs. The correlation values found for validity compare well with those reported in previous studies. For example, the developers of the Keele Pain Recorder app reported correlations of 0.79 for pain intensity and 0.60 for disability measured by the app through the question, “What is the interference of pain in the activities of the home, leisure or work,” and on paper with the 36-item Short-Form Health Survey [23]. Another app, Pain Monitor, was developed to assess pain intensity, disability, and catastrophizing and used the same instruments in its validation process as were used in this study. The authors reported a strong correlation for both pain intensity (*r*=0.71) and pain interference (*r*=0.67) when comparing the results obtained with the app and those obtained with the paper version of the Brief Pain Inventory [24].

In terms of test-retest reliability, the AvaliaDor mobile app presented excellent reliability for pain location (ICC 0.98); good reliability for pain phenotype (ICC 0.90), pain intensity (ICC 0.86), disability (ICC 0.84), and pain catastrophizing (ICC 0.76); and moderate reliability for fear of movement (ICC 0.65). These reliability values were identical to those of the paper questionnaires that served as the basis for the app questions [32,36]. However, the values of the SEM and MDD were relatively high, except those corresponding to the number of painful body sites. A difference between 2 measurements can only be considered to represent a true change in the patient’s condition if it is larger than the MDD [16]. These findings suggest that the ability to detect small changes in the patient’s condition over time may be affected. This may reflect the characteristic oscillatory behavior of chronic pain over time [45].

The mobile app AvaliaDor was considered to have good usability, which we believe is related to having taken a user-centered approach during its development [46]. The process of development included both physiotherapists and patients with pain from the very beginning—taking into account the users’ needs and expectations regarding what the app should assess and how it should look and work (ie, user requirements)—in an interactive process that ended only when users were satisfied with the app. In addition, the development of the app was carried out by a multidisciplinary team comprised of 1 individual with expertise in information technologies and 2 musculoskeletal physiotherapists, thereby involving pain specialists from the conception phase of the app until its final version was produced. Usability assessment employed a mixed methods approach using indicators of performance (eg, time used to complete a full pain assessment using the app), interviews, and validated questionnaires, in line with recommendations [47]. The lack of inclusion of users and health professionals in the development process of digital solutions targeting the health care field is one of the limitations cited by several systematic reviews [14,48,49].

### Study Limitations and Strengths

This study has limitations that must be considered. The data reported in this study were from 2 consecutive assessments only and further research is needed to assess the mobile app during prolonged use in a real-world context. Another limitation is that the mobile app is only available for the Android operating system. The strengths of the study are related to the use of the biopsychosocial model of pain assessment during the design of the app, the involvement of potential users from the beginning of the app development process, and the robust assessment of the app’s usability, reliability, and validity.

### Future Work

Future work can explore the long-term use of the AvaliaDor app in routine clinical pain management contexts and for different clinical pain conditions (eg, low back pain, neck pain, fibromyalgia), as well as the sensitivity of the app to detect changes due to interventions. Also, this mobile app can be used to facilitate high volumes of data collection, which can be analyzed with data analytics to explore potential data patterns that can inform the assessment and management of patients with pain.

### Conclusions

A mobile app—AvaliaDor—was developed to assess the intensity, location, and phenotype of pain; the associated disability; and the issues of pain catastrophizing and fear of movement. It was developed in close collaboration with patients and physiotherapists from a user-centered perspective and was shown to be usable, valid, and reliable to assess pain from a biopsychosocial perspective in a heterogeneous group of patients with pain. Future work can explore the long-term use of AvaliaDor in clinical contexts and its advantages for the assessment and management of patients with pain.

## Abbreviations

ICC: intraclass correlation coefficient
IMMPACT: Initiative on Methods, Measurement, and Pain Assessment in Clinical Trials
MDD: minimal detectable difference
MDD95: minimal detectable difference with a confidence level of 95%
mHealth: mobile health
SEM: standard error of measurement
